# Twenty years of Gendicine® rAd-p53 cancer gene therapy: The first-in-class human cancer gene therapy in the era of personalized oncology

**DOI:** 10.1016/j.gendis.2023.101155

**Published:** 2023-10-31

**Authors:** Li Qi, Guiqing Li, Peipei Li, Hongwei Wang, Xiaolong Fang, Tongchuan He, Jingjing Li

**Affiliations:** a Affiliated Hospital of Weifang Medical University, School of Clinical Medicine, Weifang Medical University, Weifang, Shandong 262700, China; b Jinming Yu Academician Workstation of Oncology, Affiliated Hospital of Weifang Medical University, Weifang, Shandong 262700, China; c Decording Therapeutics Corp, Shangha 200000, China; d Yangkun Biogroup Co., Ltd, Nanjing, Jiangsu 210002, China; e The University of Chicago, Chicago, IL 60290, USA

**Keywords:** Gendicine, Gene therapy, p53 mutation, Recombinant p53 adenovirus, *TP53*

## Abstract

Genetic mutations in *TP53* contribute to human malignancies through various means. To date, there have been a variety of therapeutic strategies targeting p53, including gene therapy to restore normal p53 function, mutant p53 rescue, inhibiting the MDM2-p53 interaction, p53-based vaccines, and a number of other approaches. This review focuses on the functions of *TP53* and discusses the aberrant roles of mutant p53 in various types of cancer. Recombinant human p53 adenovirus, trademarked as Gendicine, which is the first anti-tumor gene therapy drug, has made tremendous progress in cancer gene therapy. We herein discuss the biological mechanisms by which Gendicine exerts its effects and describe the clinical responses reported in clinical trials. Notably, the clinical studies suggest that the combination of Gendicine with chemotherapy and/or radiotherapy may produce more pronounced efficacy in slowing tumor growth and progression than gene therapy/chemotherapy alone. Finally, we summarize the methods of administration of recombinant human p53 adenovirus for different cancer types to provide a reference for future clinical trials.

## Introduction

**Figure 1:**
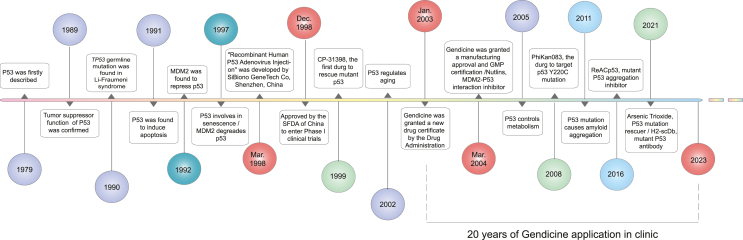
Milestones of TP53 gene targeting therapy. Since p53 was first described in 1979, its biological function has been widely and intensively investigated. In 1989, the tumor suppression function of p53 was confirmed. In the following year, TP53 germline mutation was found in Li-Fraumeni syndrome. MDM2-p53 interaction is great meaningful for drug target. Also, the drugs for mutant p53 rescue are developed. “Recombinant human p53 adenovirus injection” (Gendicine) was developed in 1998 by SiBiono GeneTech Co, Shenzhen, China, and has been applied in clinic since it was approved by Drug Administration in 2003.

According to a report from the World Health Organization, cancer has become one of the top contributors to premature mortality in most countries. The global burden of cancer continues to increase.^1^ Tumorigenesis can be driven by the alteration of one or more genes, including via gene amplification, mutation, and gene fusion. Gene therapy promotes the production of desirable or therapeutic proteins and holds great promise in cancer treatment because the therapeutic effects of the expressed proteins are highly specific. However, the cancer cell-specific delivery of genes still poses some challenges.^2^ Over the past 20 years, it has been acknowledged that gene therapy, in particular *TP53* gene therapy, shows great potential for treating even the most challenging cancers.^3^ We listed the milestones of *TP53* gene intervention therapy to show an overview since 1979 when the p53 protein was first described (Fig. 1).

As a biomarker used in the diagnosis, management, and treatment of several cancers, *TP53* is a famous tumor suppressor gene entitled the “guardian of the genome”.^4^ The protein encoded by the *TP53* gene, p53, was first described as part of a complex with the SV40 large tumor antigen in 1979.^5^ Although many other tumor suppressors play an important role in slowing down the onset and malignant progression of cancer, the p53 family members have attracted extensive attention in cancer biology.^6^, ^7^, ^8^

The expression of *TP53* is triggered by a variety of cellular stressors, including irradiation-induced DNA damage, hyperthermia, cytotoxic drugs, and oncogenic stress.^9^, ^10^, ^11^, ^12^ A multitude of biological processes, including cell cycle arrest, apoptosis, cell senescence, cell differentiation, angiogenesis, cell migration, metabolism, and DNA repair, are regulated by the wild-type p53 protein.^13^, ^14^, ^15^, ^16^ TP53 mutations are detected in around 60% of cancer cases according to a pan-cancer analysis of the TCGA database17. Mutant *TP53* can be detected in the early stages of many cancers, such as lung, skin, head and neck, and esophageal cancers,^18^, ^19^, ^20^ which is helpful for the early diagnosis of these diseases.^21^ To date, there have been a variety of therapeutic strategies targeting p53, including gene therapy to restore normal p53 function, mutant p53 rescue, inhibiting the MDM2-p53 interaction, p53-based vaccines, and a number of other approaches.^22^, ^23^, ^24^, ^25^

In this review, we focus on the functions of *TP53* and discuss the aberrant roles of mutant p53 in various types of cancer. Adenovirus-mediated p53 gene therapy using Gendicine has made tremendous progress in the field of gene therapy and illustrates the utility of targeting p53. We herein discuss the biological mechanisms by which Gendicine exerts its effects and describe the clinical responses reported in clinical trials. Notably, the clinical studies suggest that the combination of Gendicine with chemotherapy and/or radiotherapy may produce more pronounced efficacy in slowing tumor growth and progression than gene therapy/chemotherapy alone. Finally, we summarize the methods of rAd-p53 administration for different cancer types to provide a reference for future clinical trials.

## Biological functions of the p53 tumor suppressor

Genetic variations in *TP53* contribute to human malignancies in different ways.^26^^,^^27^ A variety of putative p53-mediated tumor suppression mechanisms have been described, such as inhibiting cell growth,^28^^,^^29^ reducing metastasis,^30^ regulating metabolic switching,^31^ remodeling the cancer immune microenvironment,^32^ and inducing cancer cell apoptosis,^33^, ^34^, ^35^, ^36^ autophagy,^37^ ferroptosis,^38^ and cellular senescence.^39^ Since the first study was published indicating that p53 triggers cell death, multiple mechanisms of apoptosis induced by p53 have been reported.^35^ p53 can initiate apoptosis by transcriptional activation, direct protein signaling (protein–protein interaction or another activity), or both. Interestingly, the pro-apoptotic effects of cytoplasmic p53 are not dependent on transcription. However, nuclear p53 transcriptional regulation significantly contributes to cytoplasmic p53 activity.^40^ Under pro-apoptotic conditions, p53 quickly migrates to the mitochondria and operates as a BH2-only protein, as a direct activator of Bax and/or Bak.^41^ After p53 undergoes mitochondrial translocation, it directly participates in the intrinsic apoptotic pathway by interacting with members of the Bcl-2 family of multi-structural domains (pro- and anti-apoptotic), thereby inducing mitochondrial outer membrane permeabilization.^42^^,^^43^ This evidence points to the crucial role of *TP53* in tumor suppression via induction and regular apoptosis.

Importantly, p53 also plays a central role in mediating cell cycle arrest. It is believed that p53, p16, and Rb are all associated with G1/S regulation. It was also revealed that p53 promotes the expression of the p21 protein and then inhibits the G1 to S phase transition, preventing cells from entering the DNA synthesis phase and causing cell division and growth inhibition. The CDK/cyclin complex has a direct cell growth inhibitory effect on the Rb protein, which is phosphorylated and loses its impact on G1 phase arrest, while Rb phosphorylation prevents cells from moving from the G1 phase to the S phase.^44^ One study showed that in human glioblastoma multiforme, the small molecule 2-phenol (THTMP)^45^ targets genes specific to the p53 signaling pathway, leading to a reduction in cyclin-dependent kinase protein 1, cell cycle protein A2, and cell cycle proteins E1 and E2 in glioma cells, resulting in DNA damage and cell cycle arrest in the G1/S phase. The p53 protein has also been shown to promote cell arrest in the G2/M phase after DNA damage. Other p53 target genes, such as p21, 14-3-3σ, and cdc25C may be involved in blocking the G2/M transition.^46^ The anti-oncogenic function of p53 is mainly achieved by its negatively regulating cell division and growth during cell proliferation, preventing the cell cycle from undergoing unrestricted proliferation. Following DNA damage, p53 up-regulates the expression of genes involved in cell cycle arrest and DNA repair (leading to cell survival). Various factors affect whether a cell will enter a p53-mediated apoptosis pathway or undergo cell cycle arrest.^47^

It is well known that p53 and its isoforms are also important inducers of cellular senescence.^48^, ^49^, ^50^ In addition to its clear role as a transcription factor, it has been proposed that p53 has additional functions, a notion stemming from the observation that p53 can regulate different target genes in response to diverse stress signals.^51^ Although it is still unclear which features of the tumor suppressor p53 are critical for tumor suppression and surveillance, exploiting these factors can contribute to paving the road for the tumor suppressor gene *TP53* to become a promising target for tumor gene therapy.^1^^,^^2^

## *TP53* mutations in cancer

According to the International Agency for Research on Cancer database (http://p53.iarc.fr/), *TP53* is among the most frequently mutated genes in human cancers.^3^ Different p53 mutation types are caused by different mechanisms and may differentially contribute to the malignant development of tumors. *TP53* mutations have been documented in a variety of tumor types, including colon (60%), gastric (60%), breast (20%), lung (70%), brain (40%), and esophageal (60%) cancers, with missense mutations in *TP53* being the most common (present in an estimated 75% of cases). Due to the improved protein stability and subsequent accumulation of the mutant protein, *TP53* with missense mutations can be regarded as an oncogene that promotes tumor growth, although with limited oncogenicity.^4^ Other mutations include frameshift insertions and deletions (9%), nonsense mutations (7%), and silent mutations (5%); nonsense mutations lead to premature termination codons and truncated expression of inactive p53 proteins, which may lead to p53 defects through the nonsense-mediated decay pathway.^5^ Intrinsic mutagenesis, loss of trans-activating activity, and to a lesser extent, dominant–negative activity, are the main drivers that determine the tumor phenotype. There has also been limited data suggesting that there is an acquisition of oncogenic activity (gain of function) by p53 mutants.^4^

*TP53* gene mutations stimulate hematopoiesis and tumor metastasis, promoting a more aggressive phenotype for the tumor. Moreover, *TP53* mutations have an impact on both the tumor's response to treatment and its survival following exposure to a variety of stresses. By studying mutations in the *TP53* germline associated with cancer syndromes, it was discovered that MDM2 and AKT1 were potential regulators of *TP53* because these proteins showed reduced interactions with the mutant p53 and there was a reduction in p53 degradation. Notably, the mutated proteins showed a gain-of-function activity that was not merely due to *TP53* loss.^6^ There is growing evidence that many of the gain-of-function effects of mutp53 depend on its ability to bind and inactivate the p53-associated proteins p63 and p73, wherein the p53 gain-of-function enhances cellular self-renewal and promotes the expression of cancer stem cell-associated genes, significantly contributing to invasion, metastasis, and chemo-resistant survival/multidrug resistance through activation of these genes.^7^ To reduce the clinical impact of mutant *TP53*, new therapeutic approaches have been developed to act on potential modifiers of *TP53*, and gene therapy has become one of the most important elements of molecularly targeted therapy for tumors.^8^^,^^9^ Additionally, since wild-type *TP53* regulates multiple genes that control tumor cell growth, replacing a defective p53 gene can affect multiple genes and pathways that are critical for the malignant phenotype. The tumor-associated mutant p53 protein not only loses the protective functions of wild-type p53 but also acquires a new oncogenic function independent of whether wild-type p53 is also present.^10^^,^^11^ The high frequency of p53 mutations in tumors has driven a series of research efforts to develop tumor-targeting strategies for p53 mutations. Studies have confirmed that a variety of small molecule compounds and peptide drugs can restore the wild-type activity of p53 mutants by altering their spatial conformation and folding pattern, or by promoting the degradation of mutant p53 protein to inhibit its oncogenic activity and ultimately the tumor growth.^12^, ^13^, ^14^ Identifying the mutations in somatic *TP53* may suggest new strategies to predict cancer development and progression and allow for the development of more effective medical treatments. The following sections will highlight the progress made in anti-tumor drug research targeting mutant p53.

## *TP53* as a cancer therapeutic target

Intense efforts to develop drugs that could activate or restore the p53 pathway have been ongoing since its discovery, and several agents have been evaluated in clinical trials. However, most of these efforts have met with limited success. Few p53 drug development programs have reached the advanced clinical trial stage, and to date, the US FDA has not approved any drugs targeting *TP53* directly. Studies have shown that the inhibition of mutant proteins to restore the function of wild-type p53 and the promotion of p53 mutant protein degradation are two of the most promising treatment strategies for p53 mutant tumors.^15^ The mutant p53 protein differs significantly from the wild type in terms of its spatial conformation and folding pattern and is unable to bind to the target gene promoter in a specific manner. As a result, the primary method for restoring the activity of the p53 transcription factor is to alter the high-level structure of the mutant p53 protein. It has been discovered that a variety of small molecules and peptides can interact with p53 mutants or related molecules through various pathways to cause modifications in the molecular conformation of mutant p53 proteins and change their folding patterns to the wild type, thus restoring the DNA binding ability and transcription factor activity of the mutant proteins. Additional approaches have focused on inhibiting mutant protein expression and degrading p53 mutants. It was suggested that mutations in the transactivation domain or DNA binding domain lead to different cellular localization patterns and protein binding partners, which may contribute to the molecular characterization of the cancers that carry them.^16^ These small molecules include Cys-targeting compounds, Zn^2+^chelators, peptides, and other types of compounds.^15^ CP-31398, a styryl quinazoline analog, was the first small molecule shown to reactivate mutant p53. Mechanistic studies suggested that cysteine modification plays a role in this compound's mechanism of action, and it apparently exerts its effects by protecting wild-type p53 from thermal denaturation.^17^, ^18^, ^19^ PRIMA-1 is a mutant p53 rejuvenator that acts as a selenoprotein thioredoxin reductase 1 inhibitor that binds to the sulfhydryl group of the DNA binding domain of the p53 mutants R175H and R273H.^20^^,^^21^ By regulating the redox state of cysteine, the mutant proteins are restored to the wild-type conformation and subsequently exhibit tumor suppressor activity.

## Targeting the MDM2-p53 interaction for cancer therapy

Targeting mutant p53 for cancer treatment can also be accomplished by encouraging the degradation of mutant p53 protein. In tumors that maintain wild-type p53 expression, the most widely used p53-targeted therapies involve inhibition of p53 degradation via small molecule inhibitors of the p53-MDM2 interaction, p53 gene therapy, and drug therapies that induce p53, in addition to drugs that act as chaperones by binding to mutated p53 and restoring its normal activities.^22^, ^23^, ^24^, ^25^

**Figure 2:**
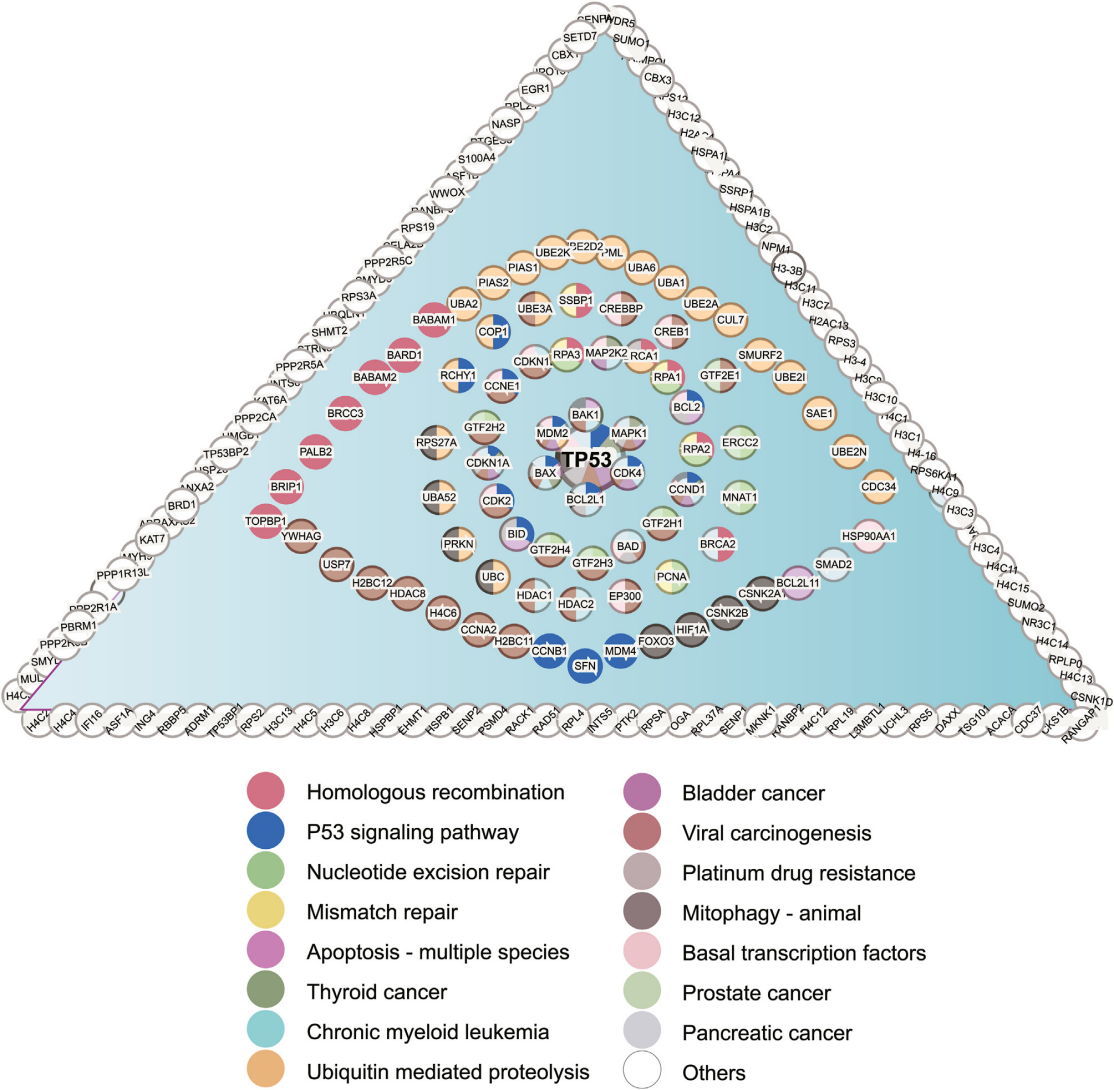
The p53 interacted protein network. P53 (center) interacts with multiple proteins and involves in many distinct pathways. Each node represents a gene. Each node directly or indirectly interacts with TP53. The pathways are depicted with different colors. For one node, multiple color coating suggest that it is involved in multiple distinct pathways. Top 15 enriched KEGG pathways are depicted. Figure is generated based on String (https://cn.string-db.org/).

One breakthrough in the field was the development of the MDM2 inhibitor, Nutlin,^26^ which was the first small molecule inhibitor of the p53-MDM2 interaction. Currently, a variety of small molecules and peptides that target the p53-MDM2 interaction or pathways have been shown to inhibit tumor growth through sustained activation of p53 target gene(s), inducing cell cycle arrest and apoptosis, and/or by reducing the resistance of cancer cells to radiotherapy (Fig. 2).

## Gendicine: a recombinant adenovirus expressing TP53 (rAd-p53) as the first-in-human cancer gene therapy

The recombinant adenoviral TP53 (rAd-p53, Gendicine; China Shenzhen SiBiono GeneTech Co., Ltd., Shenzhen, China) aims to restore the normal function of wild-type *P53*.^27^^,^^28^ Gendicine is a recombinant replication-competent human type 5 adenovirus that can create an adenoviral vector and expresses normal, functional p53 protein. The type 5 adenovirus lacks the E1 region (adenovirus oncoprotein), instead possessing a human wild-type TP53 expression cassette.^29^ Intra-arterial infusion, intravenous infusion, perfusion, and intra-tumoral injection are some of the rAd-p53 delivery routes. The first demonstration that the recombinant adenoviral p53 could inhibit tumorigenicity was reported by Zhang et al.^30^ Both preclinical and clinical studies subsequently showed that intra-tumoral injection of Ad5CMV-p53 leads to the expression of wild-type TP53 in various tumor tissues, resulting in the selective death of cancer cells.^31^^,^^32^ Based on these observations, a gene therapy clinical trial was performed by Clayman et al for the treatment of 17 patients with progressive head and neck squamous carcinoma.^33^ Recombinant adenoviruses are the most frequently used viral vectors in gene therapy,^34^^,^^35^ and rAd-p53 is particularly efficient against a variety of cancers because of its great specificity for tumor cells.^28^^,^^36^, ^37^, ^38^, ^39^

In 2003, China became the first country to approve this p53 adenovirus-based gene therapy drug (Gendicine) to be marketed for the treatment of human head and neck squamous cell carcinoma.^40^, ^41^, ^42^ This rAd-p53 is a weakened adenovirus that binds to the coxsackie-and-adenovirus receptor on tumor cells and penetrates cells selectively through receptor-mediated endocytosis, which may result in intracellular overexpression of wild-type p53, leading to p53-mediated tumor regression.^43^ Furthermore, this substance may stimulate the immune system to mount a cytotoxic T-lymphocyte immunological response against tumor cells, activate natural killer cells to exert anti-cancer "bystander effects", and suppress the expression of several oncogenes.

Gendicine is a biological therapy that can be administered by minimally invasive intra-tumoral injection, as well as by intracavitary or intravascular infusion. The *TP53* gene is usually formed by recombination of a modified type 5 or type 2 adenovirus gene with the wild-type human p53 gene. This adenoviral vector can effectively boost anti-tumor humoral and cellular immunity by producing a variety of neurological factors, hormones, and cytokines. This process is likely accomplished by regulating the neuroendocrine-immune system as a whole. By introducing exogenous *TP53* gene expression, the specific anti-tumor effects result in apoptosis or cell stasis with the rapidly dividing tumor cells while leaving normal cells largely unaffected. Recombinant viral particles and highly expressed p53 proteins can effectively trigger the body's anti-tumor immune response, and local injection can cause T lymphocytes and other cancer-killing cells to gather in tumor tissues. The p53 protein can also play a "bystander effect" on tumor cells through cellular transmission and regulation of the immune system.

## Clinical responses of Gendicine therapy in human cancers

In the 20 years since its introduction, tremendous progress has been made in identifying and developing clinical therapeutic trials for Gendicine in patients with a variety of cancers, including lung cancer, head and neck cancer, liver cancer, and cervical cancer.^44^ There are several advantages associated with rAd-p53 administration, including a high efficiency of gene transfer, with nearly 100% transduction efficiency, and it can transduce different types of cells in different human tissues. It also has high safety and controllability, as well as easy storage and transportation. It was estimated that nearly 30,000 people had been treated with Gendicine by 2013. The treatment resulted in a 30%–40% complete response rate and a 50%–60% partial response rate, for a cumulative response rate of over 90%. Notably, Gendicine also has few adverse effects and mild cytotoxicity to non-cancer cells.^41^

Although the State Food and Drug Administration of China has so far only approved Gendicine® for the treatment of head and neck cancers, others may eventually gain approval. Mutations in *TP53* are the most frequent genetic alterations detected in cancers.^3^^,^^45^ The p53 protein is pivotal in maintaining the genetic integrity after DNA damage, and alterations in the p53 pathway, including mutations in the *TP53* gene, greatly increase the probability of tumor formation.

Gendicine introduces the wild-type p53 (wt-p53) gene into cancer cells via a modified adenoviral vector, causing the wt-p53 protein to be expressed as a new gene.^46^ Gendicine has been shown to be safe and effective in the treatment of different malignancies in both preclinical and clinical trials.^47^ The extensive basic research on the p53 gene has facilitated the clinical application of Gendicine. In turn, the clinical application of Gendicine has contributed to our understanding of the functions of p53. Gendicine has become a useful tool for scientists and clinicians to collaborate on a variety of clinical studies. We have developed a database that compiles data on gene therapy clinical trials conducted in China from official agency sources, academic publications, conference presentations, and posters graciously given to us by individual scientists or trial sponsors. The findings suggest that rAd-p53 treatment is safe, practical, and very successful in treating not only human head and neck squamous cell carcinoma,^36^^,^^37^ but also other cancers such as liver cancer,^48^^,^^49^ lung cancer,^50^^,^^51^ ovarian cancer,^38^ and breast cancer. Based on available data evaluating various clinical treatment options, the data suggest that gene therapy has synergistic effects with conventional agents such as chemotherapy and/or radiotherapy and that combination treatments with Gendicine tend to provide higher response rates than standard therapies using any of the agents alone. The published clinical study data for Gendicine are summarized below (references for clinical data are shown in the supplementary file).

## Head and neck cancer

Head and neck cancer occurs in the mouth, nose, throat, larynx, sinuses, or salivary glands, and has the sixth highest incidence and seventh highest mortality rate among Chinese men.^52^^,^^53^ The most common pathological type is squamous carcinoma, with Epstein–Barr virus infection (for nasopharyngeal carcinoma), tobacco use, and alcohol use being the main causes. Radiotherapy has long been the mainstay of treatment for patients with head and neck tumors, traditionally using a stage-dependent strategy where all patients with the same TNM stage receive the same treatment.^54^ However, multidisciplinary sequential therapies, including surgery, radiotherapy, cisplatin-based chemotherapy, molecularly targeted therapies, and combination immunotherapy are commonly used. Despite the variety of therapies available, there is still an urgent need for new approaches to more effectively treat head and neck carcinoma to improve the prognosis of patients. Therefore, attempts have been made to introduce the wild-type p53 gene to treat head and neck tumors, and good results have been achieved in clinical trials.^55^

**Figure 3:**
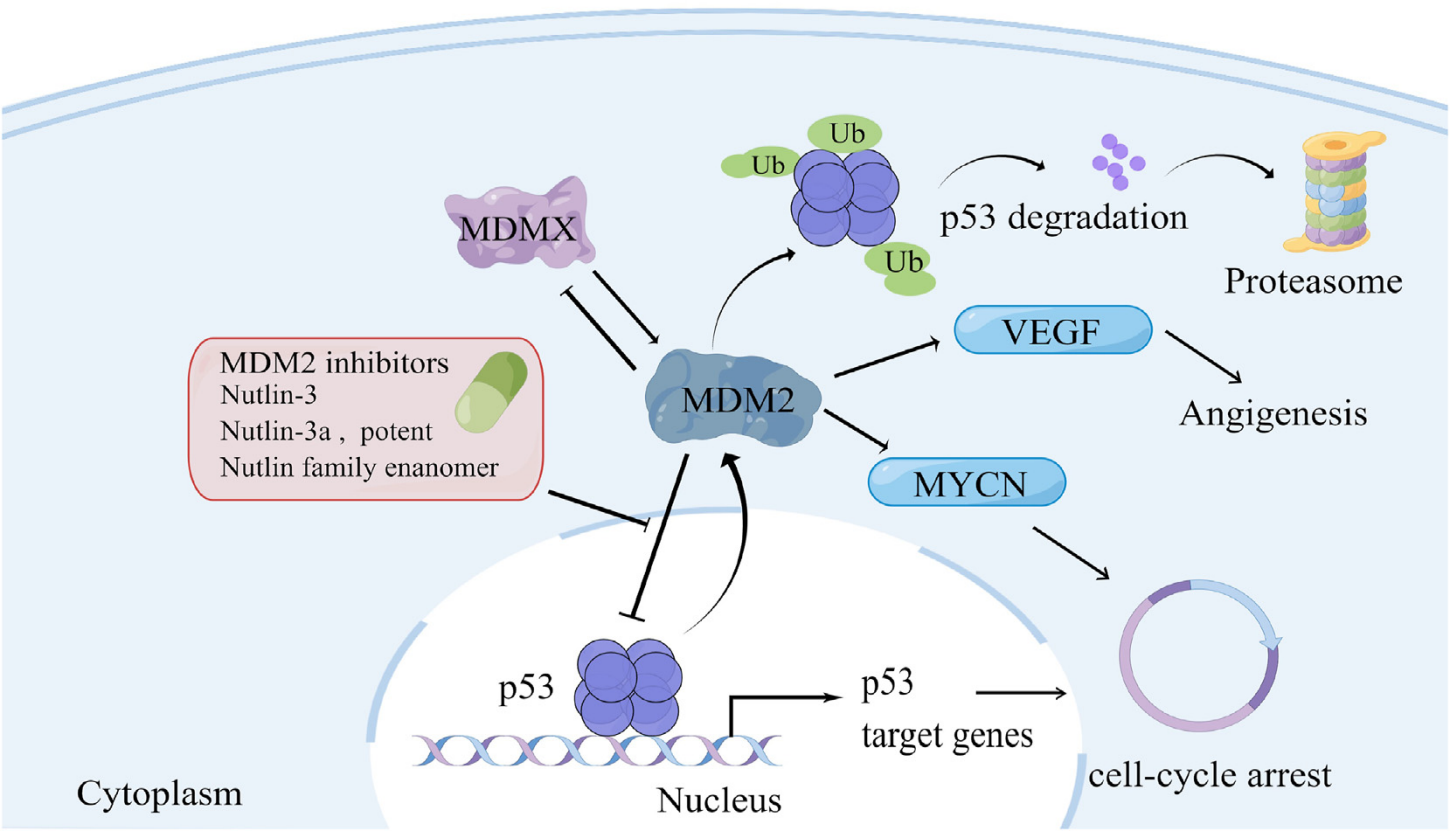
Presentive p53 functions and the drugs targeting P53-MDM2 complex. MDM2 protein binds to the N-terminal transactivation domain of p53, inhibiting its transcriptional activity as a tumor suppressor. Those drugs that inhibit MDM2 and MDM2-p53 interaction contribute to p53 normal function rescue.

A total of 688 patients with head and neck cancers have been treated with rAd-p53 injections. As indicated in the Shenzhen SiBionoGeneTech database, the complete response, partial response, stable disease, and progressive disease rates (based on the RECIST criteria) were 30%, 54%, 12.5%, and 3.3%, respectively (Fig. 3A), with most patients receiving combination therapy (rAd-p53 + chemotherapy and/or radiotherapy).

A total of 239 patients experienced adverse reactions. The most common negative effect was a self-restricting fever of degree I/II with a temperature of approximately 38 °C. Ninety-nine (13% of the total) patients experienced fever, 33 (4.7%) patients experienced gastrointestinal reactions, 29 (4.1%) patients experienced bone marrow suppression, and 14 (2.0%) patients had elevated liver enzyme levels. A very small number of patients experienced influenza-like symptoms (Fig. 3B).

## Gynecological cancers

Cervical cancer is the leading cause of cancer death in women, and ovarian cancer is the fourth leading cause of cancer death in women and the most lethal gynecologic malignancy.^56^ Since ovarian and cervical cancers have relatively high rates of p53 mutations in comparison to other types of cancer, Gendicine has also been widely used to treat these cancers. High-grade plasma ovarian cancer (the most malignant and common subtype of ovarian cancer) is characterized by *TP53* mutations, and cancer genome sequencing studies have found that more than 96% of high-grade plasma ovarian cancers have mutations in the TP53 coding region.^57^^,^^58^ Approximately 70% of patients diagnosed with ovarian cancer will present at stage III or IV.^59^ A combination of modern surgical resection and cytotoxic chemotherapy can produce a complete clinical response in 70% of patients. However, most of these patients will experience recurrence or develop drug resistance.^60^ Therefore, p53 gene therapy opens new frontiers for the treatment of ovarian cancer and may reduce the risk of recurrence or resistance. Currently, the clinical application of rAd-p53 injection in ovarian cancer is limited to patients with advanced and resistant diseases. For patients with advanced cervical cancer, intra-tumoral injection of rAd-p53 in conjunction with radiotherapy dramatically increases the rate of complete response (Fig. 3A).

In a clinical study of 317 patients with cervical cancer who received chemotherapy or radiotherapy followed by local intra-tumoral injections of Gendicine, the complete response, partial response, stable disease, and progressive disease rates were 47%, 44%, 7%, and 1%, respectively, by the end of treatment (Fig. 3A). There were 205/317 (65%) patients who developed a fever, and all recovered without intervention. A total of 117 patients had gastrointestinal reactions, 97 patients had bone marrow suppression, 23 patients had pain at the injection site, and 26 patients had urological reactions. No other serious related side effects occurred. These results show that systemic or topical application of rAd-p53 is safe, well tolerated by patients, and has some efficacy. The late side effects brought on by radiation were not made worse by rAd-p53 administration. Replacing the normal p53 gene with a viral vector has been suggested to inhibit and reverse the tumor's malignant phenotype and cause radiosensitization, resulting in a unique method for changing the tumor phenotype. These findings support the use of rAd-p53 in combination with radiotherapy. Thus, combining rAd-p53 and radiotherapy is safe and biologically active and may increase the survival rate of cervical cancer patients.

## Lung cancer

Lung cancer is the malignancy with the highest mortality and morbidity rates in most countries. Men are more likely than women to be diagnosed with lung cancer (including tracheal cancer), and lung cancer makes up 70.4% of all cancer diagnoses in men.^52^ *TP5*3 has been identified as a promising target for the treatment of lung cancer, particularly non-small cell lung cancer.^50^^,^^61^

The *TP53* gene is altered in approximately 90 % of small-cell lung cancer cases and approximately 50% of non-small-cell lung cancer cases. The majority (70%–80%) of mutations are missense point mutations, with most of these affecting the DNA binding structural domain (exons 5–8), with occasional pure deletions.^4^^,^^62^

RAd-p53 is a genetically engineered modified exogenous p53 gene transfer vector that results in high-quality thymectomy and is commonly employed in the clinical treatment of lung, head, and neck cancer on the Chinese mainland.^40^^,^^51^ A previous study suggested that conventional radiotherapy and continuous chemotherapy have poor local control in non-small cell lung cancer, with only 15%–20 % local control over 1–2 years. Adding recombinant human p53 adenovirus injection improves the radiosensitivity of lung adenocarcinoma, and rAd-p53 plus cisplatin chemotherapy resulted in a significantly higher short-term efficacy than cisplatin alone in treating malignant pleural or abdominal effusions. However, these studies had small sample sizes and focused mainly on local effects.^51^

The complete response, partial response, stable disease, and progressive disease rates among the patients in the Gendicine combination treatment group were 3.2% (17/530), 18.5% (98/530), 32% (170/530), and 12.3% (65/530), respectively, with an efficiency rate of 21.6% (Fig. 3A). These patients all received intra-tumoral injections of Gendicine, followed by radiotherapy. Following therapy, 218 patients (41.1%) had a fever, 133 patients (25.0%) had flu-like symptoms, and 174 patients (32.8%) experienced gastrointestinal issues (Fig. 3B). In this study, there were also a few reported cases of bloody coughing, psychological illnesses, and joint and muscular discomfort, but these may not have been caused by the administration of rAd-p53 but rather by the patient's underlying disease.

## Liver cancer

Primary carcinoma of the liver is a common malignancy of the digestive system that affects tens of thousands of people in China each year and is mainly caused by chronic hepatitis B virus infection. Traditional therapies like surgical resection are ideal for treating early-stage liver cancer, but alternative therapies must be used when a patient refuses surgery or when their condition is complicated by cirrhosis or chronic viral hepatitis. Although transcatheter hepatic artery chemoembolization (TACE) is often used for unresectable advanced hepatocellular carcinoma,^63^ it is of limited value because of the tumor's silent onset, rapid infiltration of the vasculature, poor prognosis, and complications from cirrhosis. The use of Gendicine in combination with TACE was considered a treatment option for hepatocellular carcinoma based on the clinical experience using it to treat other advanced cancers.^64^^,^^65^

**Figure 4:**
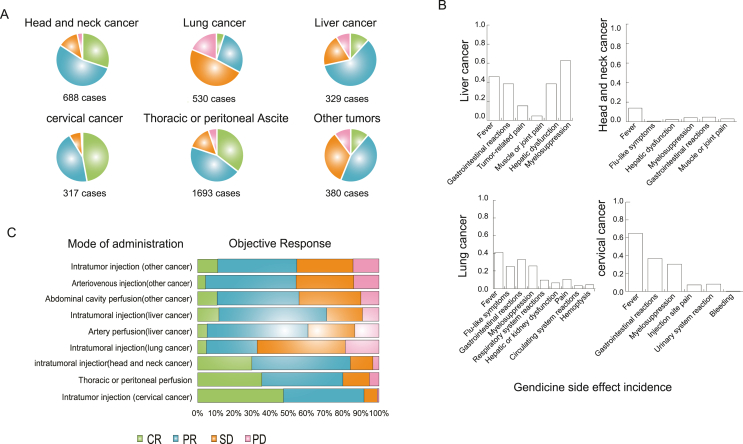
Clinical application summary of Gendicine. **(A)** Objective responses of Gendicine application in different cancer types. CR, complete response; PD, progressive disease; PR, partial response; SD, stable disease. **(B)** Side effect incidence of Gendicine in different cancer types. **(C)** Objective responses when Gendicine was injected with different modes.

Results showed that the complete response, partial response, stable disease, and progressive disease rates were 12%, 61%, 20%, and 9%, respectively, for the rAd-p53 treatment group (Fig. 4A). Febrile symptoms were again the most reported adverse effects. Of the 1026 patients in the TACE plus gene therapy group, 389 (37.9%) had a fever, 243 (23.7%) had myelosuppression, and 91 (8.8%) had mass-related pain, or muscle or joint pain that often subsided (Fig. 4B). No other serious gene therapy-related complications were encountered. These findings indicate that advanced hepatocellular carcinoma can be treated safely and effectively with rAd-p53 gene therapy in combination with TACE.

## Other types of cancer

Ad-p53 has also been used alone or in combination with radiotherapy, chemotherapy, thermotherapy, or other treatment options for other types of advanced cancer, including prostate cancer, soft tissue sarcoma, thyroid cancer, malignant glioma, and pancreatic cancer.^66^, ^67^, ^68^ Treatment with rAd-p53 has been reported to be safe and typically offers clinical benefit, even though the method of treatment varied across studies.

## Safety and efficacy of adenovirus-p53 gene therapy

rAd-p53 infection into cells is a one-time event, and the viral debris that is present after the infection is unable to reproduce within the cells, and the adenoviral DNA is no longer able to combine into the host cell genome, so it is not genotoxic to humans. Long-term survival studies in several cancer types showed that, compared with the usual treatment regimen alone, the Gendicine combination regimen offers a longer progression-free survival.^69^ Although p53 gene therapy drugs are still in clinical trials, and very few drugs have been marketed so far, the combination of adenovirus and radiotherapy for tumor treatment has the following advantages: i) reversing the resistance of tumor cells to radiotherapy; ii) synergistic effects to improve the efficacy; iii) killing residual tumor cells, prolonging disease-free survival, and improving the survival rate; iv) reducing the toxic side effects of radiotherapy and improving the quality of life by allowing for less intensive treatment.^70^

## Administration of adenovirus-p53

As noted above, a variety of delivery methods have been used for rAd-p53, with most showing significant efficacy, and intra-tumoral injection has been the most common route of delivery used in clinical studies approved by the China Food and Drug Administration.^71^^,^^72^ To ensure that the drug is expressed at a high enough concentration to maximize the efficacy of Gendicine, intravenous drips, arterial interventions, and thoracic and intraperitoneal infusions have also been considered (Fig. 4C). Direct intra-tumoral injection is not suitable for patients with widespread cancer or metastatic disease but is applicable for most solid tumors.

## Combination therapies using Gendicine and chemotherapy

Although most of the above studies discussed combination treatment with radiation therapy, Gendicine has been combined with chemotherapy for the treatment of various cancers. Patients who received the combination of rAd-p53 and chemotherapy showed a higher survival rate than those treated using standard regimens.^44^^,^^51^^,^^73^^,^^74^ Recombinant human p53 adenovirus can be injected into the tumor tissue in an interventional manner to infect and transfect tumor cells with the TP53-carrying virus, followed by local injection of chemotherapeutic agents such as bleomycin or 5-fluorouracil and administration of systemic intravenous chemotherapy. The chemotherapeutic drugs cause DNA damage to tumor cells and then wild-type p53 induces tumor cell apoptosis, resulting in sequential and systemic anti-tumor effects.^75^ Qu et al reported the results of four patients with high-grade serous ovarian cancer treated with Gendicine plus chemotherapy and showed that the combination therapy could effectively control tumor lesions, prolong the patients' survival, and improve their quality of life.^75^ Chen et al reported on the combined application of rAd-p53 and chemotherapy in patients with gastric cancer, indicating that rAd-p53 alone or in combination with oxaliplatin inhibited gastric cancer cell growth and enhanced the tumor sensitivity to oxaliplatin. A gradual decrease in the expression of apoptosis-related Bcl-2 protein was observed, suggesting that the anti-tumor effects of rAd-p53 and oxaliplatin were mediated by a mechanism that promoted apoptosis in gastric cancer cells.^73^ Thus, the combination of gene therapy (rAD-p53) and chemotherapy produced higher complete response rates than either treatment alone.

## Treatments combining Gendicine and immunotherapy

The combination of rAd-p53 with immunotherapy is beneficial for patient survival and tumor disease control. Sipuleucel-T is a novel autologous cellular immunotherapy used to treat advanced prostate cancer. Researchers generated a p53-dendritic cell vaccine and evaluated the feasibility of combining this vaccine with rAd-p53 gene therapy. Their results showed that this combination therapy could be a viable new treatment for advanced prostate cancer, regardless of the status of *p53*.^76^ Unfortunately, transfection into dendritic cells is not easy. Retroviruses, liposomes, and calcium phosphate have been used, but transduction is inefficient. Adenoviruses have been shown to provide better transduction rates into dendritic cells.

Furthermore, the combination of rAd-p53 with anti-PD-1 antibody induced a significantly higher number of tumor-infiltrating lymphocytes, including activated CD8^+^ and CD107a^+^ cytotoxic T lymphocytes.^77^^,^^78^ To date, many programs aiming at enhancing the immune response against cancer through gene therapy have been or are being carried out worldwide (*e.g.*, gene therapy through vaccines or direct cytokines or co-stimulatory molecules). It was also found that the combination of rAd-p53 with lymphokine-activated killer cell immunotherapy was more effective than this kind of immunotherapy alone for the treatment of head and neck cancer. rAd-p53 infection was shown to increase the cytotoxicity of lymphokine-activated killer cells against H891 cells, as well as increase the mRNA expression levels of UL16 binding proteins in H891 cells and tumor necrosis factor-α in lymphokine-activated killer cells.^79^ Whether the theories developed in animal models can be successfully applied to the treatment of human cancers requires further work. However, approaches combined with vaccine therapy will likely be most successful, particularly those aimed at reversing the immune dysregulation associated with cancer. It is hoped that some of these methods will soon enable cancer patients to live longer and with a higher quality of life.^76^

## Conclusions and future directions

In recent years, with the developments made in molecular biology, especially gene therapy, the application of gene-based drugs for cancer therapy has become an important clinical research trend. Gene therapy using adenoviral p53 has emerged as a potentially safe and effective treatment for many types of cancer. Nevertheless, there are still some problems that need to be solved. For example, following repeated administration of rAd-p53 injection, the expression of coxsackie-and-adenovirus receptors on the surface of tumor cells decreases, leading to a decrease in endocytosis of the p53 gene, thus leading to resistance. Further work is also needed to determine how to better combine rAd-p53 injection with conventional therapies such as radiotherapy, chemotherapy, and biologically targeted therapy. Additionally, there are new formulations of rAd-p53, such as controlled-release agents and extended-release agents, and it is necessary to determine the best timing, route, and dose of rAd-p53 to obtain the most potent effects with fewer adverse events.

Gendicine has been used for almost 20 years and was the first gene therapy product to be approved for cancer treatment. Data collected from preclinical and clinical studies have provided insight into the mechanism of action of Gendicine. However, we believe that more systematic studies are needed to further understand its therapeutic effects and to optimize its clinical application. For example, Gendicine appears to have synergistic effects with many traditional cancer treatments, including chemotherapy, radiotherapy, and thermotherapy. Our findings promise to broaden the clinical indications for Gendicine, the composition, manufacture, and clinical use of which are protected by established patents in China and around the world. However, large randomized clinical trials are still needed for validation. The relationship between rAd-p53 and long-term survival should also be explored in the future. Gene therapy has made great strides in China, but there is still a long way to go and a great deal of hard work to be done, requiring continued efforts from a wide range of researchers. As both basic and clinical research becomes more advanced, the future will yield more results that will be of great value in determining the future direction of gene-based medicine.

## Conflict of interests

The authors declare that the research was conducted in the absence of any commercial or financial relationships that could be construed as a potential conflict of interest.

## Funding

This work is supported by the 10.13039/501100001809National Natural Science Foundation of China (No. 82104289), the Yuandu Scholar Grant of Weifang City (Shandong, China) to JJL, and the Shandong Provincial Health Commission of China (No. M−2022053).
