# Supplementary material for: Twenty years of Gendicine® rAd-p53 cancer gene therapy: The first-in-class human cancer gene therapy in the era of personalized oncology
Source: Genes Dis. 2023 Oct 31;11(4):101155. doi: 10.1016/j.gendis.2023.101155 (PMC10958704; doi:10.1016/j.gendis.2023.101155)
Supplement: Multimedia component 1 [file mmc1.docx]

**Clinical data references^1-204^**

1. 贾平. 雷帕霉素联合重组人p53腺病毒注射液治疗卵巢癌的效果分析. *现代医学与健康研究电子杂志.* 2018;2(11):35.

2. 李维, 杨拴盈, 李恩孝, et al. 随机、开放、平行对照、多中心评价重组人腺病毒P53治疗恶性浆膜腔积液的临床研究. *中华肺部疾病杂志(电子版).* 2016;9(06):604-608.

3. 程金科, 林晨, 牟巨伟, 张雪艳, 邢嵘, 吴旻. 野生型p53基因重组体腺病毒介导的肿瘤抑制作用. *自然科学进展.* 1999(06):27-33.

4. 郭洪涛, 刘彤华, 高洁. 重组腺病毒介导的野生型p53对人胰腺癌细胞凋亡的诱导作用. *中华病理学杂志.* 1998(03):34-37.

5. 白雪源, 车凤翔, 李劲松, et al. 重组腺病毒介导的p16与p53基因联合转移对人肺癌细胞系H358生物学行为的影响. *中华病理学杂志.* 2000(05):33-37.

6. 赵敏, 肖绍文, 杨静贤, 张珊文, 吕有勇. 重组腺病毒-p53基因治疗患者基因变异和血清抗体检测及其临床意义. *中华医学杂志.* 2005(49):3495-3498.

7. 莫宏强, 赵凤芹, 刘晶. 重组腺病毒p53治疗老年恶性胸腔积液的临床研究. *中国老年学杂志.* 2009;29(13):1605-1607.

8. 崔荣花, 王美清, 毛山山, 程小珍, 元建华, 李兴. 重组人腺病毒注射液对胃癌细胞SGC-7901的细胞周期的影响. *中国临床药理学杂志.* 2020;36(18):2829-2831+2838.

9. 倪成亮. *重组人腺病毒介导p53基因治疗人涎腺腺样囊性癌的体外实验研究* [硕士], 福建医科大学; 2010.

10. 王翔, 张有为, 孙三元. 重组人腺病毒-p53注射液联合顺铂治疗恶性胸腹腔积液的疗效观察. *徐州医学院学报.* 2014;34(11):810-813.

11. 谢忆山. *重组人腺病毒p53联合盐酸表阿霉素抑制人胃癌细胞SGC-7901的实验研究* [博士], 武汉大学; 2011.

12. 和庆章, 王桂良, 谭松龄, et al. 重组人腺病毒P53联合放疗和单独放疗治疗中晚期恶性肿瘤有效性和安全性的Meta分析. *中国现代医生.* 2021;59(33):96-101.

13. 司勇锋. *重组人腺病毒P53联合放化疗治疗中晚期鼻咽癌的基础及临床应用研究.*

14. 余慧茜, 吴朝晖, 金益曼. 重组人腺病毒p53注射液腹腔内滴注治疗癌性腹水的护理. *安徽医药.* 2008(07):669-670.

15. 马洁韬. *重组人腺病毒p53注射液提高肺腺癌放射敏感性的体外实验研究* [硕士], 中国医科大学; 2007.

16. *重组人腺病毒p53注射液.*

17. 马洁韬, 郑伟, 邹华伟. 重组人腺病毒p53提高肺腺癌放射敏感性的实验观察. *中国医科大学学报.* 2007(04):424-426.

18. 和庆章, 王桂良, 邱萍, et al. 重组人腺病毒P53和碘125粒子调节Bax和Bcl2对裸鼠胆管癌移植瘤增殖和凋亡的影响. *中国现代医生.* 2021;59(29):44-48+193.

19. 史颖. *重组人腺病毒p53(rAd-p53)注射液对鼻咽癌细胞放射增敏作用的研究* [硕士], 大连医科大学; 2010.

20. 张金桃, 黄晓艳, 刘春红, 蒋青玉, 司勇铎, 夏辉. 重组人P_(53)腺病毒联合放化疗治疗中晚期鼻咽癌患者的护理. *护士进修杂志.* 2013;28(18):1687-1688.

21. 解春文, 解春艳. 重组人-p53腺病毒注射液. *中外医疗.* 2008(17):21-22.

22. 刘泽法. *重组人p53转染淋巴瘤源性树突状细胞的抗肿瘤免疫效应* [硕士], 兰州大学; 2008.

23. 吕汪霞, 苏丹, 冯建国, 牟瀚舟, 顾琳慧, 马胜林. 重组人p53腺病毒逆转非小细胞肺癌顺铂耐药的体外研究. *肿瘤基础与临床.* 2008(05):373-376.

24. 刘泽法, 汤华, 宋飞雪, 曾鹏云, 岳玲玲, 张连生. 重组人p53腺病毒转染淋巴瘤源性树突状细胞的抗肿瘤免疫效应. *中国实验血液学杂志.* 2012;20(03):592-597.

25. 刘泽法, 汤华, 宋飞雪, 曾鹏云, 岳玲玲, 张连生. 重组人p53腺病毒转染淋巴瘤源性树突状细胞的抗肿瘤免疫效应. *现代生物医学进展.* 2011;11(06):1087-1092.

26. 王静, 宋瑾, 卫刚, 任扩军, 戚晓东. 重组人p53腺病毒诱导乳腺癌细胞MCF-7凋亡的作用. *中国医刊.* 2014;49(04):39-42.

27. 敖敏, 何刚, 梁传余. 重组人p53腺病毒药物对人喉癌细胞的抑制实验. *现代预防医学.* 2007(06):1034-1035.

28. 张欣, 胡毅, 汪进良, et al. 重组人p53腺病毒腔内灌注治疗胸腹腔恶性积液34例疗效观察. *解放军医学院学报.* 2013;34(07):671-672+692.

29. 朱蓉, 陈亮, 孟自力, 洪永青. 重组人p53腺病毒联合顺铂治疗NSCLC合并胸腔积液临床研究. *临床肺科杂志.* 2011;16(06):896-898.

30. 殷南昌, 王晓东, 刘维. 重组人p53腺病毒联合顺铂治疗NSCLC合并恶性胸腔积液的疗效观察. *中国药房.* 2010;21(12):1120-1122.

31. 汪进良, 焦顺昌, 胡毅, 李瑾昱. 重组人p53腺病毒联合顺铂对肺腺癌A549细胞基因表达的影响. *中国医学科学院学报.* 2010;32(04):383-388+480.

32. 孔研, 曲范杰. 重组人p53腺病毒联合表阿霉素抑制胃癌细胞的作用. *中国实用医药.* 2014;9(25):253-254.

33. 唐友明, 田洪刚, 张来安, 金江林. 重组人p53腺病毒联合肝动脉栓塞化疗术治疗原发性肝癌的临床研究. *中国肿瘤临床与康复.* 2016;23(12):1424-1427.

34. 肖书萍. 重组人p53腺病毒联合肝动脉化疗栓塞治疗肝癌患者的护理. *护理学杂志.* 2009;24(09):33-34.

35. 王晚萍, 周云. 重组人p53腺病毒联合紫杉醇对胃癌细胞的作用. *医药论坛杂志.* 2007(08):1-3.

36. 郑小莉, 王婷婷, 付敏, 周健, 刘福民. 重组人P53腺病毒联合紫杉醇对宫颈癌HeLa细胞的抑制作用及其机制. *中国肿瘤生物治疗杂志.* 2013;20(02):192-196.

37. 孙京, 钱海利, 王海娟, 汪进良, 董伟伟, 胡毅. 重组人p53腺病毒联合温热顺铂不同用药方式下对人结肠癌细胞株的抑制作用. *中国现代手术学杂志.* 2014;18(02):81-85.

38. 王东, 杨志祥, 张沁宏, et al. 重组人p53腺病毒联合治疗晚期恶性肿瘤的临床观察. *临床肿瘤学杂志.* 2008(10):896-900.

39. 王建功, 王晓红, 杨俊泉, 李国欢, 胡万宁. 重组人p53腺病毒联合放化疗治疗局部晚期非小细胞肺癌. *贵阳医学院学报.* 2014;39(02):225-228.

40. 陈光侠, 郑丽红, 刘世育, 何晓华, 陆敬华, 韩新臣. 重组人p53腺病毒联合奥沙利铂对胃癌细胞BGC-823的生长抑制作用. *江苏医药.* 2012;38(05):551-554.

41. 常娜, 吴韦炜, 徐成胜, 钱立庭. 重组人p53腺病毒联合化疗治疗颈部转移癌的临床疗效观察. *中国医学创新.* 2013;10(17):3-4.

42. 崔红梅, 关崇丽, 刘青, 李莲英. 重组人p53腺病毒联合化疗对复发性卵巢癌临床疗效的评价. *中国肿瘤生物治疗杂志.* 2014;21(04):450-454.

43. 刘积良, 田耕, 金花, 朱志兵, 隋捷. 重组人p53腺病毒联合5-氟尿嘧啶治疗恶性腹腔积液. *实用临床医学.* 2008(02):18-20.

44. 杨立群, 张国志. 重组人p53腺病毒经肝动脉化疗栓塞术治疗中晚期肝癌疗效观察. *河北医药.* 2013;35(11):1664-1665.

45. 张政, 翁敬锦, 兰桂萍, et al. 重组人p53腺病毒注射液逆转鼻咽癌化疗耐药的研究. *实用医学杂志.* 2012;28(03):385-388.

46. 王莉. *重组人p53腺病毒注射液逆转胃癌耐药的实验研究* [硕士], 辽宁医学院; 2011.

47. 张林, 王季堃, 王莉. 重组人p53腺病毒注射液逆转人耐药胃癌MGC-803细胞耐药性的体外试验研究. *中国药房.* 2012;23(13):1182-1184.

48. 邱峰. *重组人p53腺病毒注射液质量稳定性的研究* [硕士], 华南理工大学; 2012.

49. 赵凤翎, 罗社文, 毛积分, 宋晓萍, 刘明贺, 许莉. 重组人p53腺病毒注射液联合顺铂胸腹腔内注射治疗恶性胸腹水疗效观察. *武警医学.* 2014;25(01):48-50.

50. 董磊. 重组人p53腺病毒注射液联合顺铂治疗肺癌致胸腔积液100例. *中国药业.* 2013;22(09):91-92.

51. 赵伟珠, 王季堃, 李巍, 张秀丽. 重组人p53腺病毒注射液联合顺铂治疗肺癌所致胸腔积液的临床研究. *癌症.* 2009;28(12):1324-1327.

52. 姚淑晖, 王晓红, 张静, 熊伟, 杨俊泉. 重组人p53腺病毒注射液联合顺铂治疗肺癌所致心包积液疗效观察. *中国煤炭工业医学杂志.* 2016;19(10):1407-1410.

53. 朱言亮, 孟云. 重组人p53腺病毒注射液联合顺铂治疗肺癌合并恶性胸腔积液的临床观察. *实用癌症杂志.* 2011;26(01):87-88.

54. 翟凤钰, 许燕艳, 王芳, 漆齐贵, 岳莉. 重组人p53腺病毒注射液联合顺铂治疗恶性腹水的疗效和安全性. *山东医药.* 2011;51(45):61-62.

55. 王月, 蔡哲, 成建, 刘维, 王亚帝, 哈敏文. 重组人p53腺病毒注射液联合顺铂治疗95例恶性胸腔积液. *广东医学.* 2010;31(22):2988-2990.

56. 王婷婷, 韩春侠, 郑小莉, et al. 重组人p53腺病毒注射液联合顺铂对宫颈癌HeLa细胞增殖及VEGF表达的影响. *徐州医学院学报.* 2012;32(02):120-123.

57. 刘素艳, 姚雪. 重组人p53腺病毒注射液联合顺铂化疗治疗恶性胸腹水临床观察. *现代预防医学.* 2011;38(17):3565-3566+3568.

58. 张瑞娟, 汪宏斌. 重组人p53腺病毒注射液联合顺铂化疗治疗恶性胸腹水64例临床观察. Paper presented at: 第五届中国肿瘤学术大会暨第七届海峡两岸肿瘤学术会议、国际肿瘤细胞与基因治疗学会会议、第二届中日肿瘤介入治疗学术会议2008; 中国河北石家庄.

59. 薛艳军, 林丽红, 高雁荣, 马媛, 韩金利, 程勇. 重组人p53腺病毒注射液联合顺铂与紫杉醇治疗晚期宫颈癌的疗效及安全性分析. *临床医学工程.* 2021;28(04):437-438.

60. 郭成安, 李会兰, 魏莉. 重组人P53腺病毒注射液联合调强放疗与同步化疗治疗中晚期宫颈癌的临床疗效分析. *河北医药.* 2016;38(08):1206-1209.

61. 曲怡梅, 廖国清, 王红梅, 刘鹏辉, 李亮亮, 解国清. 重组人p53腺病毒注射液联合胸腔热灌注化疗治疗肺癌所致胸腔积液的临床研究. *疑难病杂志.* 2011;10(08):594-596.

62. 郭成安. 重组人P53腺病毒注射液联合立体适形放疗与热疗治疗中晚期宫颈癌的近期疗效观察. *河北医药.* 2015;37(06):875-877.

63. 王勇利, 兰桂萍, 司勇锋, et al. 重组人p53腺病毒注射液联合治疗对鼻咽癌组织中KAI1和CD44 v6表达的影响. *中国耳鼻咽喉颅底外科杂志.* 2014;20(02):96-100.

64. 王晓红, 王建功, 张静, 胡万宁, 张瑞娟. 重组人p53腺病毒注射液联合放疗治疗鼻咽鳞癌的临床观察. *现代中西医结合杂志.* 2012;21(09):924-925+934.

65. 郭亚茹, 许明娜, 方苗, 杨松, 辛勇. 重组人p53腺病毒注射液联合放疗或同步放化疗治疗宫颈癌疗效及安全性的Meta分析. *现代肿瘤医学.* 2022;30(14):2584-2590.

66. 俞泽阳, 范我, 吴锦昌, et al. 重组人p53腺病毒注射液联合放射治疗对人淋巴瘤细胞的影响. *辐射防护.* 2008(03):177-183.

67. 俞泽阳. *重组人p53腺病毒注射液联合放射治疗对人淋巴瘤疗效的实验研究* [博士], 苏州大学; 2008.

68. 陈惠玉, 史桂芳, 陈香英. 重组人p53腺病毒注射液联合放射治疗中晚期宫颈癌的护理. *海峡药学.* 2009;21(08):141-143.

69. 范宇飞, 秦苑, 李定纲. 重组人P53腺病毒注射液联合局部热疗与化疗治疗晚期胰腺癌的效果观察. *中国当代医药.* 2017;24(13):84-87.

70. 杨俊泉, 王晓红, 郑国红, 赵洪焕, 胡万宁. 重组人p53腺病毒注射液联合同期放化疗治疗老年T4N0-2M0局部晚期非小细胞肺癌26例临床分析. *中国煤炭工业医学杂志.* 2013;16(10):1586-1589.

71. 陈锐, 仝晓敬, 马跃, 陶亚楠, 王治海. 重组人P53腺病毒注射液联合化疗治疗晚期恶性肿瘤的近期疗效评价. *临床和实验医学杂志.* 2014;13(16):1326-1329.

72. 张丹, 李恩孝, 李曾. 重组人p53腺病毒注射液联合化疗治疗晚期宫颈癌的临床疗效观察. *实用癌症杂志.* 2019;34(04):681-683.

73. 王晓红, 杨俊泉, 李国欢, 胡万宁, 王建功. 重组人p53腺病毒注射液联合化疗治疗恶性胸腔积液临床观察. *中国煤炭工业医学杂志.* 2014;17(02):188-191.

74. 曹信杰, 张颖, 李会兰, 王会平, 王志勇, 魏莉. 重组人p53腺病毒注射液联合化疗治疗恶性体腔积液的疗效观察. *中国药房.* 2005(23):1809-1810.

75. 孙秀梅, 高峰, 李贵新, 白冰玉, 马长庚, 张敏. 重组人p53腺病毒注射液联合化疗在胃癌患者中的应用. *山东医药.* 2010;50(43):85-86.

76. 郑小莉, 刘永利, 周彩霞, 吴小进, 刘太峰. 重组人P53腺病毒注射液联合化疗在早期巨块型宫颈癌术前治疗中的效果. *中国医药导报.* 2016;13(20):84-87.

77. 李莹. *重组人p53腺病毒注射液联合伊立替康诱导结肠癌细胞株HT-29凋亡的实验研究* [硕士], 辽宁医学院; 2012.

78. 蒋明, 马华兵, 杨瑞梅, 凡治国, 任超. 重组人P53腺病毒注射液联合介入治疗肝癌18例分析. *中国医疗前沿.* 2012;7(10):39+17.

79. 杨曼春. 重组人P53腺病毒注射液结合介入治疗妇科盆腔恶性肿瘤. *医药论坛杂志.* 2010;31(15):177-178.

80. 王辉, 马永全, 杜芳, 马若巾, 王梅, 张利娟. 重组人p53腺病毒注射液治疗难治性癌性胸水近期疗效. *实用医药杂志.* 2010;27(02):131.

81. 罗倩茹. 重组人p53腺病毒注射液治疗肺癌恶性胸腔积液的疗效观察及护理. *中国医疗前沿.* 2010;5(17):82-83.

82. 何婉, 许瑞莲, 朱莲玉, et al. 重组人p53腺病毒注射液治疗晚期卵巢癌的疗效观察. *中国肿瘤临床与康复.* 2015;22(07):782-785.

83. 周宗远, 刘霞. 重组人p53腺病毒注射液治疗恶性胸腔积液的临床疗效观察. *四川医学.* 2013;34(05):695-696.

84. 曲荣锋, 郭东瑞, 夏大文. 重组人P53腺病毒注射液治疗恶性胸腔积液临床疗效观察. *激光杂志.* 2007(06):96-97.

85. 陶静. 重组人P53腺病毒注射液治疗头颈肿瘤的护理体会. Paper presented at: 第十四次中国口腔颌面外科学术会议2018; 中国重庆.

86. 徐红艳, 焦惠民, 郭亚丽, 李凤, 许志鹏. 重组人p53腺病毒注射液治疗口腔粘膜白斑对患者肿瘤坏死因子TNF-α的影响. *肿瘤药学.* 2018;8(03):400-402+410.

87. 张恩欣. 重组人p53腺病毒注射液局部治疗耐药舌癌1例及分析. *中国肿瘤临床.* 2010;37(10):545.

88. 仲智勇, 时保军, 周辉, 王文博. 重组人p53腺病毒注射液对肾母细胞瘤细胞增殖、凋亡及自噬的影响. *中国药房.* 2017;28(07):889-892.

89. 冯晓峰, 郑永志, 周春柳, 孔祥毓, 李淑德, 孙卫东. 重组人p53腺病毒注射液在原发性肝癌介入化疗中的应用价值. *世界华人消化杂志.* 2013;21(15):1437-1441.

90. 何志江, 温坚, 吕榆莲, 张卓云, 邱振华. 重组人p53腺病毒注射液介入治疗原发性肝癌临床疗效观察. *昆明医科大学学报.* 2015;36(08):87-90.

91. 董磊. 重组人p53腺病毒注射液与顺铂序贯胸腔灌注联合热疗治疗肺癌胸腔积液临床观察. *中国肿瘤临床与康复.* 2013;20(08):855-857.

92. 李会兰, 曹信杰, 张颖, et al. 重组人P53腺病毒注射液、DDP、5-Fu腹腔灌注联合热疗治疗恶性腹腔积液临床观察. *河北医药.* 2010;32(14):1862-1863.

93. 刘蓉蓉, 姬长友, 陈继川. 重组人p53腺病毒注射液(今又生)联合放疗治疗复发性鼻咽癌疗效观察. *山东大学耳鼻喉眼学报.* 2010;24(05):13-16.

94. 曹信杰. *重组人P53腺病毒注射液(rAd-P53)治疗癌性胸、腹水临床研究.*

95. 韩波, 史善伟, 李茂泉, 鲁立光, 李龙江, 李一. 重组人p53腺病毒注射液(rAd-p53)治疗头颈部恶性黑色素瘤的分子机制及临床研究. Paper presented at: 第十一次全国口腔颌面——头颈肿瘤学术会议暨2017山东省口腔医学会口腔颌面外科分会学术年会暨山东省口腔颌面外科高层论坛暨山东省口腔医学会口腔颌面一头颈肿瘤分会成立大会2017; 中国山东济南.

96. 史善伟, 李茂泉, 鲁立光, 李龙江, 李一. 重组人p53腺病毒注射液(rAd-p53)治疗头颈部恶性黑色素瘤的分子机制及临床研究. Paper presented at: 第十三次全国口腔颌面外科学术会议暨中华口腔医学会口腔颌面外科专业委员会成立30周年纪念活动2016; 中国辽宁沈阳.

97. 彭朝晖. *重组人p53腺病毒注射液.*

98. 伏虹. 重组人p53腺病毒注射治疗恶性胸腔积液的护理观察. *中外医学研究.* 2015;13(32):105-106.

99. 李冬瑞. *重组人P53腺病毒注入术治疗门静脉癌栓的临床疗效研究* [硕士], 河北医科大学; 2013.

100. 林健泽, 江建明, 李振宇, 闫洪印, 孙炜. 重组人p53腺病毒治疗脊柱转移瘤的近期疗效观察. *华西医学.* 2010;25(05):883-885.

101. 赵凤芹, 张佳, 季红. 重组人p53腺病毒治疗肺腺癌恶性胸腔积液的临床疗效. *中国老年学杂志.* 2016;36(23):5921-5923.

102. 陈晓秋, 李柱, 申东兰. 重组人p53腺病毒治疗结肠腺癌腹壁转移1例. *中国肿瘤临床.* 2006(11):660.

103. 阮冬梅. 重组人p53腺病毒治疗癌性腹水的研究. *中外医学研究.* 2011;9(04):32-33.

104. 丁洪琼. 重组人p53腺病毒治疗晚期胰腺癌患者的护理. *护理学杂志.* 2008;23(24):65-66.

105. 李磊. *重组人p53腺病毒治疗恶性胸腔积液的研究* [硕士], 吉林大学; 2006.

106. 刘春雨. 重组人p53腺病毒治疗恶性肿瘤的护理进展. *护理研究.* 2012;26(08):673-675.

107. 曹晓静, 戴楠, 杨志祥, et al. 重组人p53腺病毒治疗原发性肝癌的血清p53蛋白检测及其临床意义. *肿瘤预防与治疗.* 2009;22(01):5-8.

108. 朱云霞, 尤国美, 余先萍. 重组人p53腺病毒治疗中晚期肝癌的毒副反应护理. *护理与康复.* 2012;11(09):895-896.

109. 王晚萍, 周云. 重组人p53腺病毒提高胃癌细胞对顺铂敏感性的实验研究. *实用诊断与治疗杂志.* 2007(05):331-332+335.

110. 秦成勇, 梁铁军, 蒋莹, 张才擎. 重组人p53腺病毒抑制肝癌HepG_2细胞增殖机制的研究. *山东医药.* 2005(29):7-9.

111. 刘明月, 王晚萍, 周云. 重组人p53腺病毒感染对携带不同p53状态胃癌细胞增殖和凋亡的影响. *胃肠病学和肝病学杂志.* 2012;21(08):740-742.

112. 王晚萍. *重组人p53腺病毒对胃癌细胞生长及化疗敏感性的实验研究* [硕士], 郑州大学; 2007.

113. 杨倩, 关弘, 张家庭, 李征毅. 重组人p53腺病毒对肝癌细胞作用的病理学观察. *云南医药.* 2010;31(02):136-139.

114. 段薇, 项芬芬, 高英慧, 冯一丹, 张学梅. 重组人p53腺病毒对糖尿病小鼠糖代谢的影响. *中国糖尿病杂志.* 2016;24(12):1100-1104.

115. 梁寒. *重组人p53腺病毒对人胃癌和肠癌细胞的作用及其与热疗对放化疗的增敏作用* [硕士], 天津医科大学; 2009.

116. 余培东, 方菁. 重组人p53腺病毒对中晚期肝癌免疫功能的影响. *宁夏医学杂志.* 2014;36(04):349-350.

117. 张沁宏, 向德兵, 卿毅, et al. 重组人P53腺病毒增强肝癌细胞放疗的敏感性. *中国肿瘤生物治疗杂志.* 2008(05):458-463.

118. 张沁宏, 向德兵, 卿毅, et al. 重组人p53腺病毒增强肝癌细胞放疗敏感性的实验研究. Paper presented at: 第五届中国肿瘤学术大会暨第七届海峡两岸肿瘤学术会议、国际肿瘤细胞与基因治疗学会会议、第二届中日肿瘤介入治疗学术会议2008; 中国河北石家庄.

119. 余舒亮, 王东, 张沁宏, et al. 重组人p53腺病毒增强肝癌放疗敏感性的实验研究. *第三军医大学学报.* 2009;31(09):784-787.

120. 敖敏, 何刚, 乔晓明, 梁传余. 重组人p53腺病毒基因药物抑制喉癌细胞生长的实验研究. *华西医学.* 2007(03):579-581.

121. 敖敏, 何刚. 重组人p53腺病毒基因药物对人鼻咽癌细胞的抑制实验. *西部医学.* 2010;22(09):1596-1597+1600.

122. 郑传胜, 罗仕华, 冯敢生. 重组人P53腺病毒基因经介入方法治疗肝癌的的动物实验研究. Paper presented at: 中国(第七届)肿瘤微创治疗学术大会暨世界影像导引下肿瘤微创治疗学会成立筹备大会2011; 中国广东广州.

123. 罗仕华, 郑传胜, 冯敢生. 重组人P53腺病毒基因经介入方法治疗肝癌的的动物实验研究. Paper presented at: 2010湖北省肿瘤介入治疗学术大会2010; 中国湖北武汉.

124. 罗仕华, 郑传胜, 冯敢生. 重组人P53腺病毒基因经介入方法治疗肝癌的的动物实验研究. Paper presented at: 第十一次全国中西医结合影像学术研讨会暨全国中西医结合影像学研究进展学习班2010; 中国湖北武汉.

125. 郑传胜, 罗仕华, 冯敢生. 重组人P53腺病毒基因经介入方法治疗肝癌的动物实验研究. Paper presented at: 第五届中国肿瘤学术大会暨第七届海峡两岸肿瘤学术会议、国际肿瘤细胞与基因治疗学会会议、第二届中日肿瘤介入治疗学术会议2008; 中国河北石家庄.

126. 郑传胜, 罗仕华, 冯敢生. 重组人P53腺病毒基因经介入方法. Paper presented at: 2009年世界肿瘤介入学术大会2009; 中国北京.

127. 罗仕华, 郑传胜, 冯敢生, 梁惠民, 周国锋, 夏向文. 重组人p53腺病毒基因经介入后在兔VX2肝癌中表达. *世界华人消化杂志.* 2010;18(05):437-442.

128. 来娟. 重组人p53腺病毒基因治疗腹膜后平滑肌肉瘤1例的护理体会. *解放军护理杂志.* 2005(06):100.

129. 崔红梅, 关崇丽, 李莲英, 刘青. 重组人p53腺病毒在晚期卵巢癌治疗中临床疗效及安全性评价. *现代肿瘤医学.* 2014;22(01):154-157.

130. 班永光, 唐军. 重组人p53腺病毒制品治疗原发性肝癌的进展. *医学影像学杂志.* 2006(04):408-410.

131. 汪东. *重组人p53腺病毒PLGA缓释微球磷酸钙骨水泥复合物抑癌作用的动物实验* [硕士], 蚌埠医学院; 2014.

132. 李东辉, 王海鹏, 孙华, 李超. 重组人p53基因腺病毒注射液在原发性肝癌介入治疗中的临床研究. *现代肿瘤医学.* 2015;23(22):3286-3289.

133. 周小娟, 司小敏, 王云梅, 吕建建. 重组p53腺病毒对人肺腺癌H1299细胞体内外的抑制作用. *中国肿瘤生物治疗杂志.* 2013;20(04):409-413.

134. 梁晓宁, 郭瑞军, 于泽兴, 张颖, 初玉萍, 张建军. 超声引导下经皮注射重组人p53腺病毒注射液(今又生)在治疗晚期转移性肿瘤中的应用. Paper presented at: 中国超声医学工程学会第八届全国腹部超声学术会议2010; 中国山东青岛.

135. 贡雪灏, 朱志兵, 张家庭, 李泉水, 张琪. 超声引导下瘤体内注射重组人p53腺病毒治疗晚期肝癌. *华中科技大学学报(医学版).* 2008(04):499-501+505.

136. 崔琳, 杨林. 调强放疗与化疗联合重组人P53腺病毒注射液治疗IIb期-IIIb期宫颈鳞癌的临床疗效. *临床检验杂志(电子版).* 2017;6(02):173-175.

137. 崔琳. *调强放疗与化疗联合重组人P53腺病毒注射液治疗ⅡB~ⅢB期宫颈鳞癌的临床疗效* [硕士], 安徽医科大学; 2018.

138. 牟巨伟, 林晨, 邢嵘, 王秀琴, 吴旻. 腺病毒介导野生型p53基因对人肺腺癌细胞的抑制效应. *内蒙古医学杂志.* 2002(04):291-294+377.

139. 谢琦, 杨逸铭, 吴敏仪, et al. 腺病毒介导的p53逆转结肠癌对5-FU耐药性的实验研究. *中华临床医师杂志(电子版).* 2016;10(12):1746-1752.

140. 何峰, 李帅, 朱霞霞, 杨吉成, 盛伟华, 缪竞诚. 腺病毒介导ING4或/和P53基因表达对人肺腺癌细胞的生长抑制作用. *上海交通大学学报(医学版).* 2015;35(07):953-960.

141. 李永恒, 刘长青, 徐刚, et al. 腹腔灌注“重组人p53腺病毒注射液”结合热化疗治疗癌性腹膜炎(附41例报告). Paper presented at: 中国抗癌协会肿瘤放射治疗专业委员会学术大会中美放射肿瘤协会（SANTRO）第三届学术会议2012济南国际放射肿瘤学论坛2012; 中国山东济南.

142. 秦苑, 任东, 范宇飞, 刘晓林, 李定纲. 腹腔内灌注重组人p53腺病毒注射液及顺铂结合局部亚高温热疗治疗恶性腹水观察. *实用临床医药杂志.* 2011;15(13):15-17.

143. 唐博, 谭群友, 王如文, et al. 胸腔内注射重组人p53腺病毒治疗肺癌恶性胸腔积液的临床研究. *局解手术学杂志.* 2015;24(02):192-194.

144. 朱慧利, 解平. 胃镜下瘤内注射重组人p53腺病毒治疗晚期食道癌的安全性及短期疗效观察. *实用医院临床杂志.* 2012;9(04):79-80.

145. 马小延, 俞新燕, 朱丽婵. 肺癌患者颈部淋巴结转移瘤体内注射重组人p53腺病毒的护理. *护理学报.* 2011;18(13):58-59.

146. 官泳松, 贺庆. 肝癌介入治疗中应用p53的几个要点. *介入放射学杂志.* 2008;17(11):761-763.

147. 祝朝前, 殷军. 肝动脉化疗栓塞术联合重组人p53腺病毒动脉灌注治疗肝细胞癌临床观察. *山东医药.* 2017;57(15):57-59.

148. 刘丽, 卲天朋, 杨涛, 曹建民, 卢光明, 许健. 经动脉灌注rAd-p53联合TAE治疗中晚期肝癌的疗效观察. *介入放射学杂志.* 2016;25(03):210-213.

149. 罗仕华, 郑传胜, 冯敢生, 梁惠民, 周国锋, 夏向文. 经介入重组人p53腺病毒基因在兔VX2肝癌中表达研究. Paper presented at: 2010湖北省肿瘤介入治疗学术大会2010; 中国湖北武汉.

150. 罗仕华, 郑传胜, 冯敢生, 梁惠民, 周国锋, 夏向文. 经介入重组人p53腺病毒基因在兔VX2肝癌中表达研究. Paper presented at: 第十一次全国中西医结合影像学术研讨会暨全国中西医结合影像学研究进展学习班2010; 中国湖北武汉.

151. 王建华, 张涛, 贡桑明久, 毛智军, 李文生. 经不同途径应用rAdp53对大肠癌裸鼠模型生长抑制差异性的研究. *现代肿瘤医学.* 2010;18(03):451-453.

152. 翁准, 覃天力, 谭淑瑜, 刘积良, 隋捷, 朱志兵. 瘤内注射重组人p53腺病毒治疗晚期肺癌临床试验观察. *深圳中西医结合杂志.* 2004(04):206-210.

153. 孙金杰, 司勇锋, 兰桂萍, 覃扬达, 王勇利, 翁敬锦. 瘤内注射重组人p53腺病毒对鼻咽癌组织中基质金属蛋白酶-13表达的影响及意义. *广西医学.* 2015;37(12):1723-1726.

154. 任维维. *生物免疫治疗非小细胞肺癌的系统评价和Meta分析* [硕士], 兰州大学; 2014.

155. 边晨峰, 王辉. 热灌注化学治疗联合重组人p53腺病毒治疗肝癌腹腔积液临床研究. *中国药业.* 2019;28(18):72-75.

156. 张欣. *热化疗联合重组人p53腺病毒对人肺癌细胞的作用研究* [硕士], 中国人民解放军医学院; 2013.

157. 徐祯祯, 权循凤. 放疗联合重组人P53腺病毒注射液治疗恶性肿瘤的研究进展. *安徽医学.* 2014;35(11):1621-1624.

158. 张冬娟, 杨林, 陈象逊, 汪浩. 放疗联合p53腺病毒治疗中晚期宫颈癌近期疗效分析. *安徽医科大学学报.* 2017;52(06):874-878.

159. 刘东芳, 王晓红, 刘春秋, et al. 支气管动脉灌注重组人p53腺病毒联合同期放化疗治疗局部晚期非小细胞肺癌的近期疗效及安全性. *中国肿瘤临床与康复.* 2015;22(11):1332-1335.

160. 唐博, 谭群友, 王如文, et al. 手术联合胸腔内留置重组人p53腺病毒治疗非小细胞肺癌的围手术期安全性. *中国肿瘤.* 2015;24(05):430-434.

161. 许辉茹, 冯慧晶, 杨晓玲, 张俊萍. 恶性积液局部灌药疗效观察. *中外医学研究.* 2017;15(03):14-15.

162. 张瑞娟. 恶性实体肿瘤内注射p53联合放化疗临床观察. *现代中西医结合杂志.* 2012;21(18):1966-1967.

163. 赵军伟, 张鹏, 安红丽, et al. 应用颈阔肌肌皮瓣联合重组人p53腺病毒注射液治疗牙龈癌患者的效果及对MMP-9、TIMP-1、VEGF表达的影响. *临床和实验医学杂志.* 2020;19(06):599-603.

164. 祁华琼, 黄伟明. 岭南名中医黄伟明之学术思想浅谈. *心电图杂志（电子版）.* 2020;9(2):265-266.

165. 赵健竹, 韩琤波, 邹华伟. 外源性p53基因导入不同时限后联合放疗对肺腺癌细胞A549的影响. *中国医科大学学报.* 2010;39(07):539-541+545.

166. *基因治疗药物-重组人p53腺病毒注射液.*

167. 张冬娟. *同步放化疗联合p53腺病毒治疗中晚期宫颈癌近期疗效分析* [硕士], 安徽医科大学; 2017.

168. 汪惠, 赖百塘, 李伟英, et al. 制备两种p53重组腺病毒和流式细胞仪定量外源绿荧光蛋白表达. *中国肺癌杂志.* 2010;13(05):470-476.

169. 夏敏, 占强, 郭继中, 陈涛, 王含芬. 内镜超声引导下瘤内注射rAd-p53联合吉西他滨治疗胰腺癌14例. *实用医学杂志.* 2011;27(16):2998-3000.

170. 张跃伟, 娜仁图戈, 李闯, 冀学宁. 内镜下rAd—p53注射联合动脉灌注治疗进展期胃癌. *当代医学.* 2010;16(11):171-172.

171. 阮继刚, 杨力, 张飞雄, 孟祥坤, 苗雨, 冯丽丹. 内镜下rAd-p53注射治疗中晚期胃癌和食管癌的临床效果研究. *宁夏医科大学学报.* 2012;34(11):1136-1139+1238.

172. 邢嵘, 林晨, 牟巨伟, 程金科, 张雪艳, 吴旻. 介导p53基因转移的重组体腺病毒对人肺癌细胞的抑制作用. *大连医科大学学报.* 2000(02):84-87.

173. 顾朋, 布力布·吉力斯汉, 樊喜文. 介入化疗治疗联合重组人P53腺病毒注射液对胃癌治疗的疗效分析. *解放军预防医学杂志.* 2019;37(09):180-181.

174. 张自新, 张靖, 何建莉, 折虹. 今又生联合放疗治疗晚期恶性肿瘤的近期疗效观察. *宁夏医学杂志.* 2012;34(12):1273-1274.

175. 郑永法, 戈伟, 张令, 王慧敏, 邓君健. 今又生联合化疗治疗晚期恶性肿瘤的近期疗效观察. *生物医学工程与临床.* 2011;15(01):60-63.

176. 李岩, 马海英. 今又生联合化疗治疗恶性胸腔积液的护理体会. *中国现代药物应用.* 2007(10):61.

177. 周云, 王晚萍. 人重组p53腺病毒感染p53突变mBGC-823细胞对顺铂敏感性的影响. *郑州大学学报(医学版).* 2009;44(05):948-951.

178. 高文娟, 金玉, 段招军. 人腺病毒的研究进展. *病毒学报.* 2014;30(02):193-200.

179. 张礼来, 张志琴, 周凤玲. 三甲散加减联合重组人p53腺病毒注射液治疗血瘀型喉癌前病变疗效及对血清VEGF和β_2-MG的影响. *现代中西医结合杂志.* 2017;26(36):4058-4060.

180. 都庆国, 张涛, 王建华, et al. Tf-PEG脂质体-rAdp53复合物、rAdp53腹腔内灌注治疗晚期结直肠癌合并恶性腹腔积液的临床研究. *现代肿瘤医学.* 2013;21(05):1087-1090.

181. 崔荣花, 王美清, 彭大为, et al. rAd-p53联合顺铂对胃癌细胞生长及KAI1/CD82蛋白表达的影响. *实用癌症杂志.* 2016;31(01):10-13.

182. 孙晓谦. *rAd-p53联合规范化治疗恶性胶质瘤的疗效观察* [硕士], 大连医科大学; 2013.

183. 汪海岩, 赵利红, 张敬川, 许统俭, 韩秋裕, 于大海. rAd-p53联合化疗及局部热疗治疗恶性浆膜腔积液. *中国肿瘤.* 2012;21(09):717-720.

184. 李代龙, 庞雅琪, 许新华. rAd-p53联合TACE治疗原发性肝癌有效性和安全性的Meta分析. *巴楚医学.* 2022;5(01):91-98.

185. 王建华, 纪宗正, 闫立昆. rAd-p53的抑癌作用及体外转染后抑癌蛋白的表达. *肿瘤防治研究.* 2007(03):185-188+232.

186. 张林, 王季堃, 王莉. rAd-p53注射液联合热化疗治疗老年性非小细胞肺癌胸腔积液41例. *中国老年学杂志.* 2015;35(01):213-214.

187. 陶振超, 钱立庭, 邱俊, 王明明. rAd-p53对人肝癌细胞生长作用的影响. *安徽医科大学学报.* 2013;48(04):353-356.

188. 陶振超, 邱俊, 钱立庭, 王明明, 吴爱东, 闫冰. rAd-p53对人肝癌细胞放射敏感性影响的实验研究. *安徽医科大学学报.* 2012;47(04):396-399.

189. 陶振超. *rAd-p53对人肝癌细胞放射敏感性影响的实验研究* [硕士], 安徽医科大学; 2012.

190. 王少龙, 何依群, 姜鹤群, 周艳刚, 张杰. rAd-p53基因联合放疗和热疗治疗非手术食管癌的临床观察. *海南医学.* 2012;23(07):1-3.

191. 朱朝辉, 曾甫清, 林晨, et al. p53重组腺病毒联合顺铂或三氧化二砷对人膀胱癌EJ细胞的作用. *中华实验外科杂志.* 2002(03):34-35.

192. 王新昭. *p53调节EGFR-TKI对三阴性乳腺癌细胞敏感性研究* [硕士], 济南大学; 2015.

193. 敖敏, 何刚. p53基因药物抑制人鼻咽癌生长的免疫组化研究. *四川医学.* 2009;30(08):1193-1194.

194. 杨立群, 王瑞林, 王惠, 彭玉梅. P53基因状态与应用今又生的疗效研究. *中外医疗.* 2009;28(31):16-17.

195. 崔守章, 史守良, 陶红, 张丽, 陈宝明. P53基因治疗药动静脉给药治疗原发性肝癌的疗效比较. *河北医药.* 2014;36(18):2758-2759.

196. 覃扬达, 翁敬锦, 兰桂萍, et al. p53基因治疗对鼻咽癌患者局部免疫及疗效的影响. *临床耳鼻咽喉头颈外科杂志.* 2012;26(21):980-983.

197. 周日晶, 翁敬锦, 司勇锋, et al. p53基因治疗对鼻咽癌患者CD34标记的微血管密度和血小板计数的影响. *广西医学.* 2014;36(02):187-190.

198. 史守良, 董秀敏, 胡万宁, 马龙滨, 陈宝明, 韩敏利. P53基因治疗原发性肝癌的临床研究. *现代中西医结合杂志.* 2012;21(10):1036-1037.

199. 张松涛, 李龙江. MTT法观察rAd-p53对人口腔粘膜白斑细胞增殖的影响. Paper presented at: 第五次全国口腔颌面—头颈肿瘤学术研讨会2006; 中国湖北武汉.

200. 王静, 宋瑾, 卫刚, 任扩军, 戚晓东. Ad-p53诱导乳腺癌细胞MDA-MB-231的凋亡作用. *现代肿瘤医学.* 2014;22(06):1239-1242.

201. 周承志, 欧阳铭, 李时悦, 谭获. 96例肺癌患者合并恶性胸腔积液的临床疗效观察. *中国医药指南.* 2011;9(22):5-7.

202. 熊茂婧, 毕小琴. 34例重组人腺病毒P53基因治疗颌面部恶性肿瘤的护理. *当代护士(下旬刊).* 2012(07):90-91.

203. 张长明, 左书耀, 闫瑞红. ~(32)P-胶体磷酸铬联合p53基因治疗恶性胸腔积液的临床研究. *泰山医学院学报.* 2012;33(02):92-94.

204. 邹小辉. *5型和41型人腺病毒E1B55K与E4orf6蛋白相互作用研究* [博士], 中国疾病预防控制中心; 2013.
