# Supplementary material for: Twenty years of Gendicine® rAd-p53 cancer gene therapy: The first-in-class human cancer gene therapy in the era of personalized oncology
Source: Genes Dis. 2023 Oct 31;11(4):101155. doi: 10.1016/j.gendis.2023.101155 (PMC10958704; doi:10.1016/j.gendis.2023.101155)
Supplement: Multimedia component 3 [file mmc3.docx]

Human diseases can be driven by the alteration of one or more genes, including via gene amplification, mutation, and gene fusion, et al. Gene therapy promotes the production of desirable or therapeutic proteins and holds great promise. The first gene therapy is human myoblast genome therapy (HMGT) or myoblast transfer therapy (MTT) (HMGT/MTT) for hereditary degenerative diseases, which is conducted in 1990^1^ . The donor myoblasts provide a normal nucleus into the dystrophic muscle fibres to make multinucleated heterokaryons that compensate for genetic defects. Since then, many efforts have been made in the gene therapy area. In 2003, the Chinese State Food and Drug Administration (SFDA) approved Gendicine(rAd-p53), the first cancer gene therapy product which is indicated for head and neck cancer therapy^2^. Although it goes through numerous ups and downs, gene therapy has come of age. So far, more than 40 gene therapy products have been approved globally.

TP53 is a famous tumour suppressor gene entitled the "guardian of the genome ". A variety of putative p53-mediated tumour suppression mechanisms have been described, such as inhibiting cell growth, reducing metastasis, regulating metabolic switching, remodelling the cancer immune microenvironment, and inducing cancer cell apoptosis, autophagy, ferroptosis, and cellular senescence^3,4^. The protein encoded by the TP53 gene, p53, was first described as part of a complex with the SV40 large tumour antigen in 1979^5^. TP53 is among the most frequently mutated genes in human cancers. Different p53 mutation types are caused by different mechanisms and may differentially contribute to the malignant development of tumours. TP53 mutations have been documented in a variety of tumour types, including colon (60%), gastric (60%), breast (20%), lung (70%), brain (40%), and oesophageal (60%) cancers, with missense mutations in TP53 being the most common (present in an estimated 75% of cases).

Genetic mutations in TP53 contribute to human malignancies through various means^6^ ^7^ ^8,9^. TP53 gene mutations stimulate hematopoiesis and tumour metastasis, promoting a more aggressive phenotype for the tumour. Moreover, TP53 mutations have an impact on both the tumour’s response to treatment and its survival following exposure to a variety of stresses. The tumour-associated mutant p53 protein not only loses the protective functions of wild-type p53 but also acquires a new oncogenic function independent of whether wild-type p53 is also present^10,11^. The high frequency of p53 mutations in tumours has driven a series of research efforts to develop tumour-targeting strategies for p53 mutations.

To date, there have been a variety of therapeutic strategies targeting p53, including gene therapy to restore normal p53 function, mutant p53 rescue, inhibiting the MDM2-p53 interaction, p53-based vaccines, and several other approaches. This review focuses on the functions of TP53 and discusses the aberrant roles of mutant p53 in various types of cancer. rAd-p53 (recombinant human p53 adenovirus), trademarked as Gendicine, which is the first anti-tumour gene therapy drug, has made tremendous progress in the field of cancer gene therapy. The "Recombinant Human p53 Adenovirus Injection" was developed by SiBiono GeneTech Co and first described publicly in March 1998. In December of the same year, the drug was approved by the State Food and Drug Administration (SFDA) of China to enter Phase I clinical trials. After being investigated in several clinical trials, the recombinant p53 adenovirus (rAd-P53) injection, named "Gendicine", was granted a new drug certificate by the Drug Administration in 2003. Gendicine became the first gene therapy product in the world to receive official approval and enter the commercial market as a product for the treatment of head and neck cancer.

There have now been at least 52 clinical trial protocols utilizing recombinant human p53 adenovirus products worldwide for the treatment of 26 different malignant tumors. Based on 20 years of commercial use in more than 30,000 patients and more than 30 published clinical studies, Gendicine has demonstrated an impeccable safety profile. Combination treatment with Gendicine also leads to significantly higher response rates for conventional chemotherapy and radiotherapy compared to these standard therapies alone. The continuing interaction between basic and clinical research is one of this review's most intriguing features. In the future, it is anticipated that this information will help patients by enabling the development of novel treatments.

We herein discuss the biological mechanisms by which Gendicine exerts its effects and describe the clinical responses reported in clinical trials. Notably, the clinical studies suggest that the combination of Gendicine with chemotherapy and/or radiotherapy may produce more pronounced efficacy in slowing tumour growth and progression than gene therapy/chemotherapy alone. Finally, we summarize the methods of rAd-p53 administration for different cancer types to provide a reference for future clinical trials.

However, gene therapy still faces many challenges to overcome. Safety is always the most important issue. Looking back at the history of gene therapy development, we have entered a new era, as evidenced by an expanded spectrum of clinical indications, more sophisticated approaches for gene editing, as well as more optimized vectors.

Our team focuses on precision cancer therapy and the rationality of the combined application of clinical drugs. Our final goal is to offer and plan specific care for our patients, based on particular genes, proteins, and other substances. One research effort is defining the gene targets of pan-gynaecologic tumours, especially in ovarian cancer which has a high mortality rate than others. Besides, we ask whether the drug combination is rational in cancer chemotherapy. Here the combined drugs might not be two anticancer drugs, those drugs are usually considered as “assistant drugs” worth more attention.

We have established a mature system for cancer precision therapy, from molecular mechanism research, animal models, human organoid library, and bioinformatic analysis, to clinical trials. We bring together cell biologists, clinicians, and bioengineers to achieve our goals.

References

1. Law PK, Bertorini TE, Goodwin TG, et al. Dystrophin production induced by myoblast transfer therapy in Duchenne muscular dystrophy. *Lancet.* 1990;336(8707):114-115.

2. Xia Y, Li X, Sun W. Applications of Recombinant Adenovirus-p53 Gene Therapy for Cancers in the Clinic in China. *Curr Gene Ther.* 2020;20(2):127-141.

3. Aubrey BJ, Strasser A, Kelly GL. Tumor-Suppressor Functions of the TP53 Pathway. *Cold Spring Harb Perspect Med.* 2016;6(5).

4. Levine AJ. P53 and The Immune Response: 40 Years of Exploration-A Plan for the Future. *Int J Mol Sci.* 2020;21(2).

5. Patil MR, Bihari A. A comprehensive study of p53 protein. *J Cell Biochem.* 2022;123(12):1891-1937.

6. Kastenhuber ER, Lowe SW. Putting p53 in Context. *Cell.* 2017;170(6):1062-1078.

7. Oren M, Rotter V. Mutant p53 gain-of-function in cancer. *Cold Spring Harbor Perspectives In Biology.* 2010;2(2):a001107.

8. Harford JB, Kim SS, Pirollo KF, Chang EH. TP53 Gene Therapy as a Potential Treatment for Patients with COVID-19. *Viruses.* 2022;14(4).

9. Shahryari A, Saghaeian Jazi M, Mohammadi S, et al. Development and Clinical Translation of Approved Gene Therapy Products for Genetic Disorders. *Front Genet.* 2019;10:868.

10. Mantovani F, Collavin L, Del Sal G. Mutant p53 as a guardian of the cancer cell. *Cell Death Differ.* 2019;26(2):199-212.

11. Zhang C, Liu J, Xu D, Zhang T, Hu W, Feng Z. Gain-of-function mutant p53 in cancer progression and therapy. *J Mol Cell Biol.* 2020;12(9):674-687.
